# Some characteristics of hyperglycaemic crisis differ between patients with and without COVID-19 at a safety-net hospital in a cross-sectional study

**DOI:** 10.1080/07853890.2021.1975042

**Published:** 2021-09-11

**Authors:** Arnav Shah, Andrew Deak, Shaneisha Allen, Elayna Silfani, Christina Koppin, Yaara Zisman-Ilani, Imali Sirisena, Christina Rose, Daniel Rubin

**Affiliations:** a Lewis Katz School of Medicine, Temple University, Philadelphia, PA, USA; b Section of Endocrinology, Diabetes, and Metabolism, Lewis Katz School of Medicine at Temple University, Philadelphia, PA, USA; c School of Pharmacy, Temple University, Philadelphia, PA, USA; d Department of Social and Behavioral Sciences, College of Public Health, Temple University, Philadelphia, PA, USA

**Keywords:** COVID-19, hyperglycaemic emergencies, diabetic ketoacidosis, hyperglycaemic hyperosmolar syndrome

## Abstract

**Objective:**

To compare patients with DKA, hyperglycaemic hyperosmolar syndrome (HHS), or mixed DKA-HHS and COVID-19 [COVID (+)] to COVID-19-negative (−) [COVID (−)] patients with DKA/HHS from a low-income, racially/ethnically diverse catchment area.

**Methods:**

A cross-sectional study was conducted with patients admitted to an urban academic medical center between 1 March and 30 July 2020. Eligible patients met lab criteria for either DKA or HHS. Mixed DKA-HHS was defined as meeting all criteria for either DKA or HHS with at least 1 criterion for the other diagnosis.

**Results:**

A total of 82 participants were stratified by COVID-19 status and type of hyperglycaemic crisis [26 COVID (+) and 56 COVID (−)]. A majority were either Black or Hispanic. Compared with COVID (−) patients, COVID (+) patients were older, more Hispanic and more likely to have type 2 diabetes (T2D, 73% vs 48%, *p* < .01). COVID(+) patients had a higher mean pH (7.25 ± 0.10 vs 7.16 ± 0.16, *p* < .01) and lower anion gap (18.7 ± 5.7 vs 22.7 ± 6.9, *p* = .01) than COVID (−) patients. COVID (+) patients were given less intravenous fluids in the first 24 h (2.8 ± 1.9 vs 4.2 ± 2.4 L, *p* = .01) and were more likely to receive glucocorticoids (95% vs. 11%, *p* < .01). COVID (+) patients may have taken longer to resolve their hyperglycaemic crisis (53.3 ± 64.8 vs 28.8 ± 27.5 h, *p* = .09) and may have experienced more hypoglycaemia <3.9 mmol/L (35% vs 19%, *p* = .09). COVID (+) patients had a higher length of hospital stay (LOS, 14.8 ± 14.9 vs 6.5 ± 6.0 days, *p* = .01) and in-hospital mortality (27% vs 7%, *p* = .02).

**Discussion:**

Compared with COVID (−) patients, COVID (+) patients with DKA/HHS are more likely to have T2D. Despite less severe metabolic acidosis, COVID (+) patients may require more time to resolve the hyperglycaemic crisis and experience more hypoglycaemia while suffering greater LOS and risk of mortality. Larger studies are needed to examine whether differences in management between COVID (+) and (−) patients affect outcomes with DKA/HHS.

## Introduction

The clinical presentation and outcomes of patients with COVID-19 and hyperglycaemic emergency (i.e. diabetic ketoacidosis [DKA] or hyperglycaemic hyperosmolar syndrome [HHS]) have been described [1–3]. Few studies have directly compared patients in DKA with COVID-19 to those without COVID-19 [4–6] and none have specifically studied patients at a safety-net hospital. The objective of this study was to compare the clinical characteristics, management, and outcomes of patients with DKA, HHS, or mixed DKA-HHS and a positive SARS-CoV-2 RT-PCR to negative patients with DKA/HHS from a low-income, racially and ethnically diverse catchment area.

## Patients and Methods

A cross-sectional study was conducted with patients admitted to an urban academic medical Centre in Philadelphia, PA, USA between 1 March and 30 July 2020. Data were collected by computer query and manual review of electronic health records. Selection bias was limited by enrolling all eligible patients. Patients met lab criteria for either DKA (serum bicarbonate ≤18 mEq/L, anion gap >10, ketonaemia or ketonuria, and arterial pH ≤7.30), or HHS (blood glucose >33.3 mmol/L and osmolality >320 mOsm/kg) [7]. Mixed DKA-HHS was defined as meeting all criteria for either DKA or HHS with at least 1 criterion for the other diagnosis. Diagnoses were adjudicated by an endocrinologist. Continuous variables were compared between COVID-19 positive patients [at least one positive SARS-CoV-2 RT-PCR, COVID (+)] and negative patients [all negative SARS-CoV-2 RT-PCR or not tested, COVID (−)] with *t*-tests and categorical variables were compared with chi-square. For DKA/HHS subgroup comparisons across COVID-19 status, ANOVA, chi-square, or Fisher Exact tests were used. The Temple University Institutional Review Board approved the study (number 27246). The requirement for informed consent was waived because this was a study of pre-existing retrospective data.

## Results

A total of 82 participants were eligible and stratified by COVID-19 status and type of hyperglycaemic crisis (26 COVID (+) and 56 COVID (−), Table 1). A majority of the patients were either Black or Hispanic. The distribution of hyperglycaemic emergency type was not significantly different between COVID (+) and COVID (−) groups. Compared with COVID (−) patients, COVID (+) patients were older (mean age 58.0 ± 14.3 vs 50.0 ± 18.0 years, *p* = .05), identified more as Hispanic (35% vs 15%, *p* < .05) and were more likely to have type 2 diabetes (T2D, 73% vs 48%, *p* < .01). Among those with DKA, T2D was more common in COVID (+) than in COVID (−) patients, whereas T1D was more common in COVID (-) patients (*p* = .02). Additionally, COVID (+) DKA patients had less severe ketoacidosis with a higher pH (*p* = .03) and bicarbonate (*p* < .01) and lower anion gap (*p* < .01) than COVID (−) patients. Overall, COVID (+) patients had a higher mean pH (7.25 ± 0.10 vs 7.16 ± 0.16, *p* < .01) and lower anion gap (18.7 ± 5.7 vs 22.7 ± 6.9, *p* = .01) than COVID(−) patients. COVID (+) patients were given less intravenous fluids in the first 24 h after admission (2.8 ± 1.9 vs 4.2 ± 2.4 L, *p* = .01) and were more likely to receive glucocorticoids (95% vs. 11%, *p* < .01). COVID (+) patients may have taken longer to resolve their hyperglycaemic crisis (53.3 ± 64.8 vs 28.8 ± 27.5 h, *p* = .09) and may have experienced more hypoglycaemia <3.9 mmol/L (35% vs 19%, *p* = .09). COVID (+) patients had a higher length of hospital stay (LOS, 14.8 ± 14.9 vs 6.5 ± 6.0 days, *p* = .01) and in-hospital mortality (27% vs 7%, *p* = .02).

**Table 1.** Clinical and biochemical characteristics of COVID (+) and COVID (−) patients with hyperglycaemic crisis.

Variable			COVID Positive				COVID Negative				*p* Value^c^
	All Cases^a^	All^a^	DKA^b^	HHS^b^	Mixed DKA/HHS^b^	All^a^	DKA^b^		HHS^b^	Mixed DKA/HHS^b^	
N	82	26	20 (77%)	3 (12%)	3 (12%)	56	36 (64%)		10 (18%)	10 (18%)	–
**Demographics**											
Age	52.7 ± 17.5	58.0 ± 14.3	57.0 [52.0–68.0]	69.0 [..]	48.0 [46.5–67.5]	50.0 ± 18.0	48.5 [33.0–60.0]		61.5 [52.0–71.5]	54.0 [43.0–63.8]	.05
Male, n (%)	47 (57%)	12 (46%)	11 (55%)	1 (33%)	0 (0%)	35 (63%)	21 (58%)		8 (80%)	6 (60%)	.16
Hispanic ethnicity, n (%)	16 (21%)	8 (35%)	7 (39%)	0 (0%)	1 (50%)	8 (15%)	7 (19%)		0 (0%)	1 (13%)	.05
Race, n (%)	–	–	–	–	–	–	–		–	–	.07
White	12 (15%)	2 (8%)	2 (10%)	0 (0%)	0 (0%)	10 (18%)	7 (19%)		2 (30%)	0 (0%)	–
Black	43 (52%)	11 (42%)	9 (45%)	1 (33%)	1 (33%)	32 (57%)	21 (58%)		5 (50%)	6 (60%)	–
Other/Unknown	27 (33%)	13 (50%)	9 (45%)	2 (67%)	2 (67%)	14 (25%)	8 (22%)		2 (20%)	4 (40%)	–
**Diabetes Status**											
History of diabetes, n (%)	56 (68%)	15 (58%)	12 (60%)	2 (67%)	1 (33%)	41 (73%)	27 (75%)		8 (80%)	6 (60%)	.16
Diabetes type, n(%)	–	–	–	–	–	–	–		–	–	<.01
Type 1	23 (28%)	1 (4%)	1 (5%)	0 (0%)	0 (0%)	22 (39%)	14 (39%)		2 (20%)	6 (60%)	–
Type 2	46 (56%)	19 (73%)	14 (70%)	2 (67%)	3 (100%)	27 (48%)	15 (42%)		8 (80%)	4 (40%)	–
Indeterminate	13 (16%)	6 (23%)	5 (25%)	1 (33%)	0 (0%)	7 (13%)	7 (19%)		0 (0%)	0 (0%)	–
Hemoglobin A1C, mmol/mol	95 ± 8	91 ± 9	92 [65–115]	105 [90–121]	107 [..]	99 ± 3	102 [89–119]		81 [80–130]	108 [95_121]	.31
Hemoglobin A1C, %	10.8 ± 2.9	10.5 ± 3.0	10.6 [8.1-12.7]	11.8 [10.4-13.2]	11.9 [..]	11.2 ± 2.4	11.5 [10.3-13.0]		9.6 [9.5–14.0]	12.0 [10.8–13.2]	.31
**Labs on admission**											
Beta-hydroxybutyrate, mmol/L	3.6 ± 2.4	4.4 ± 3.6	4.3 ± [0.9–6.7]	..	4.5 [..]	3.3 ± 1.8	4.5 [1.0–4.5]		1.0 [0.5–2.1]	4.5 [4.5–4.5]	.36
Urine ketones	–	–	–	–	–	–	–		–	–	.28
Not measured, n (%)	4 (5%)	1 (4%)	1 (5%)	0 (0%)	0 (0%)	3 (5%)	3 (8%)		0 (0%)	0 (0%)	–
15, n (%)	30 (37%)	8 (31%)	6 (30%)	0 (0%)	2 (67%)	22 (39%)	17 (47%)		0 (0%)	5 (50%)	–
40, n (%)	16 (20%)	4 (15%)	2 (10%)	2 (67%)	0 (0%)	12 (21%)	6 (17%)		6 (60%)	0 (0%)	–
> =80, n (%)	13 (16%)	3 (12%)	3 (15%)	0 (0%)	0 (0%)	10 (18%)	5 (14%)		3 (30%)	2 (20%)	–
Negative or trace, n (%)	19 (23%)	10 (29%)	8 (40%)	1 (33%)	1 (33%)	9 (16%)	5 (14%)		1 (10%)	3 (30%)	–
Osmolality, mOsm/kg	312 ± 27	307 ± 31	292 [286–308]	353 [341–368]	321 [321–339]	316 ± 27	299 [291–309]		339 [326–347]	332 [224–357]	.18
Creatinine, µmol/L	301 ± 507	234 ± 292	138 [87–225]	168 [121-202]	157 [136–169]	288 ± 421	135 [47–227]		202 [164–308]	209 [140–287]	.56
eGFR, mL/min/1.73 m^2^	38.2 ± 17.8	39.2 ± 18.4	42.5 [23.8–60.0]	33.0 [31.0–46.5]	38.0 [30.5–41.0]	36.9 ± 18.3	44.0 [28.5–60.0]		32.0 [20.8–45.0]	28.0 [20.5–36.0]	.60
Glucose, mmol/L	29 ± 16	29 ± 18	21 [15–32]	45 [43–53]	49 [45–50]	36 ± 21	26 [18–33]		51 [42–62]	54 [40–64]	.18
Anion gap	20.2 ± 5.1	18.7 ± 5.7	18.5 [15.5–22.0]	10.0 [10.0–15.5]	20.0 [18.5–22.5]	22.7 ± 6.9	24.0 [19.8–27.0]		15.5 [12.5–19.8]	30.0 [23.5–30.0]	.01
pH	7.20 ± 0.14	7.25 ± 0.10	7.24 [7.20–7.30]	7.37 [7.31–7.40]	7.26 [7.26–7.29]	7.16 ± 0.16	7.14 [7.06–7.27]		7.29 [7.18–7.32]	7.23 [7.11–7.26]	<.01
Bicarbonate, mmol/L	13.0 ± 5.0	15.0 ± 5.0	16.0 [13.0–17.0]	26.0 [23.0–26.0]	15.0 [14.0–15.0]	13.0 ± 7.0	12.5 [7.0–15.0]		23.0 [17.5–24.8]	8.5 [6.0–12.8]	.07
Sodium, mmol/L	133 ± 8	133 ± 8	133 [128–136]	147 [139–150]	131 [131–138]	131 ± 8	129 [127–132]		132 [128–135]	133 [126–141]	.26
Potassium, mmol/L	4.9 ± 1.1	4.8 ± 1.0	4.6 [3.9–4.9]	5.9 [5.1–6.4]	5.2 [4.7–5.5]	4.9 ± 1.3	4.4 [4.0–5.3]		5.2 [5.1–5.5]	5.1 [4.4–5.6]	0.52
**Clinical Treatment**											
Insulin given, day 1, units	79 ± 55	86 ± 62	86 [6–148]	133 [119–152]	119 [106–124]	76 ± 51	58 [16–101]		80 [65–94]	135 [66–149]	.47
Insulin given, day 1, units/kg	1.0 ± 0.7	1.0 ± 0.8	1.1 [0.1–1.5]	1.2 [1.1–1.6]	65.8 [62.4–78.3]	1.0 ± 0.7	0.8 [0.3–1.1]		1.0 [0.9–1.3]	1.8 [1.1–2.0]	.85
Primary insulin route, n (%)	–	–	–	–	–	–	–		–	–	.75
IV	60 (74%)	18 (69%)	14 (70%)	2 (67%)	2 (67%)	42 (76%)	27 (77%)		7 (70%)	8 (80%)	–
SQ	14 (17%)	5 (19%)	4 (20%)	1 (33%)	0 (0%)	9 (16%)	6 (17%)		2 (20%)	1 (10%)	–
not given insulin	7 (9%)	3 (12%)	2 (10%)	0 (0%)	1 (33%)	4 (7%)	2 (6%)		1 (10%)	1 (10%)	–
Fluids given, day 1, L	3.7 ± 2.5	2.8 ± 1.9	3.0 [1.4–4.3]	2.9 [2.5–3.5]	1.3 [1.3–1.4]	4.2 ± 2.4	4.0 [2.7–5.1]		3.6 [2.8–5.0]	1.5 [1.1–2.5]	.01
Time to resolution of DKA/HHS, h	32.9 ± 32.7	52.3 ± 64.8	31.2 [9.6–62.4]	52.8 [46.8–69.6]	57.6 [24.8–69.6]	28.8 ± 27.5	16.8 [9.0–26.4]		19.5 [12.9–27.6]	33.6 [15.5–56.3]	.09
Glucocorticoid use, n (%)	29 (89%)	23 (79%)	19 (95%)	2 (67%)	2 (67%)	6 (11%)	4 (11%)		1 (10%)	1 (10%)	<.01
Hypoglycaemia during treatment, n (%)											
<3.9 mmol/L (70 mg/dl)	19 (23%)	9 (35%)	7 (35%)	1 (33%)	1 (33%)	10 (18%)	7 (19%)		1 (10%)	2 (20%)	.09
<3.0 mmol/L (54 mg/dl)	5 (6.1%)	2 (8%)	1 (5%)	0 (0%)	1 (33%)	3 (5%)	1 (2.8%)		1 (10%)	1 (10%)	.68
<2.2 mmol/L (40 mg/dl)	0 (0%)	0 (0%)	0 (0%)	0 (0%)	0 (0%)	0 (0%)	0 (0%)		0 (0%)	0 (0%)	–
**Outcomes**											
Length of hospital stay, d	9.1 ± 10.4	14.8 ± 14.9	10.6 [7.0–19.3]	5.9 [5.3–13.3]	6.8 [5.2–9.4]	6.5 ± 6.0	4.2 [2.1–8.2]		5.6 [3.1–9.8]	5.3 [4.3–8.9]	.01
ICU stay, n (%)	58 (72%)	17 (65%)	12 (60%)	2 (67%)	3 (100%)	41 (75%)	26 (72%)		7 (70%)	8 (89%)	.39
In-hospital mortality, n (%)	11 (14%)	7 (27%)	6 (30%)	1 (33%)	0 (0%)	4 (7%)	2 (6%)		1 (10%)	1 (10%)	.02

^a^Data are *n* (%) or mean ± SD; ^b^Data are *n* (%) or median [iQR]; ^c^All COVID positive vs. All COVID negative; ".." indicates data not available.

## Discussion

Compared with COVID (−) patients, COVID (+) patients with DKA/HHS are more likely to have T2D. Despite less severe metabolic acidosis, COVID (+) patients may require more time to resolve the hyperglycaemic crisis and experience more hypoglycaemia while requiring longer hospital stays and suffering a greater risk of mortality. Whether or not the difference in clinical management in terms of intravenous fluids and glucocorticoids is associated with outcomes remains unclear.

While other studies have described the association of COVID-19 with T2D, higher LOS, and greater mortality among DKA patients,[4–6] metabolic differences from COVID (-) patients have not been found consistently. We are unaware of other studies comparing COVID (+) and COVID (−) patients on such an extensive list of variables across types of hyperglycaemic emergencies. Furthermore, this study uniquely focussed on racially and ethnically diverse patients at a safety-net hospital. These findings may not be generalizable to other populations, and the study is limited by a modest sample size drawn from a single center. Larger studies are needed to examine whether differences in management between COVID (+) and COVID (−) patients affect outcomes with DKA/HHS.

## Data Availability

The data that support the findings of this study are available from the corresponding author, DR, upon reasonable request.
